# A Heterogeneous Ensemble Approach for Travel Time Prediction Using Hybridized Feature Spaces and Support Vector Regression

**DOI:** 10.3390/s22249735

**Published:** 2022-12-12

**Authors:** Jawad-ur-Rehman Chughtai, Irfan ul Haq, Saif ul Islam, Abdullah Gani

**Affiliations:** 1 Department of Computer and Information Sciences (DCIS), Pakistan Institute of Engineering and Applied Sciences (PIEAS), Islamabad 44000, Pakistan; 2 Digital Disruption Lab, DCIS, PIEAS, Islamabad 44000, Pakistan; 3 Department of Computer Science, Institute of Space Technology, Islamabad 44000, Pakistan; 4 Faculty of Computing and Informatics, University Malaysia Sabah, Labuan 88400, Malaysia

**Keywords:** intelligent transportation systems, travel time prediction, hybridized feature space, heterogeneous ensemble learning

## Abstract

Travel time prediction is essential to intelligent transportation systems directly affecting smart cities and autonomous vehicles. Accurately predicting traffic based on heterogeneous factors is highly beneficial but remains a challenging problem. The literature shows significant performance improvements when traditional machine learning and deep learning models are combined using an ensemble learning approach. This research mainly contributes by proposing an ensemble learning model based on hybridized feature spaces obtained from a bidirectional long short-term memory module and a bidirectional gated recurrent unit, followed by support vector regression to produce the final travel time prediction. The proposed approach consists of three stages–initially, six state-of-the-art deep learning models are applied to traffic data obtained from sensors. Then the feature spaces and decision scores (outputs) of the model with the highest performance are fused to obtain hybridized deep feature spaces. Finally, a support vector regressor is applied to the hybridized feature spaces to get the final travel time prediction. The performance of our proposed heterogeneous ensemble using test data showed significant improvements compared to the baseline techniques in terms of the root mean square error (53.87±3.50), mean absolute error (12.22±1.35) and the coefficient of determination (0.99784±0.00019). The results demonstrated that the hybridized deep feature space concept could produce more stable and superior results than the other baseline techniques.

## 1. Introduction

Intelligent transportation systems (ITSs) deal with the ever-evolving nature of travel demands and ever-changing transportation infrastructures by intelligently utilizing and allocating traffic resources. Smart traffic infrastructures and artificial intelligence-based algorithms for data analysis play pivotal roles in ITSs. Smart traffic infrastructures enable us to obtain large volumes of traffic data using a wide array of devices, including handheld devices, in-vehicle navigation systems, and loop detectors, among many others. Then, data analysis algorithms help to convert this raw data into useful information that can be used to draw conclusions and inferences about traffic.

Travel time prediction (TTP) is one of the essential services in ITSs; more specifically, it assists in navigation applications and ATISs. Precise advanced traveler information systems (ATISs) make trip planning easier and allow logistic and transportation companies to operate and manage their everyday operations more efficiently.

Recently, successful data-driven approaches have been devised that formulate travel time (TT) as a pure regression task, which can directly estimate the TT of complete paths/routes using historical data by implicitly modeling traffic complexities [1,2,3]. The existing data-driven approaches can be divided into trajectory-based approaches and origin-destination (OD)-based approaches. OD-based approaches only take into account pick-up and drop-off location data and do not consider intermediate trajectories [1], while trajectory-based approaches do consider intermediate trajectories [2,3].

Another perspective is the prediction horizon of TTP studies. TTP studies have generally been grouped into three categories: short-term (5–30 min), medium-term (30 min–24 h), and long-term (more than a day) TTP [4]. One study [5] divided TTP into short-term and long-term TTP, with prediction horizons of 0–60 min and longer than a day, respectively. TTP studies have also been categorized into real-time or online TTP, as well as short-term and long-term TTP [6]: the prediction of travel time at the current time without knowing future conditions is classified as real-time TTP, short-term TTP has a prediction horizon of 0–60 min and long-term TTP has a prediction horizon of over a day. The study of short-term TTP requires the collection of traffic data within a shorter period. Historical travel time data and other exogenous factors, such as weather, calendar data, events, etc., become more important as the prediction horizon increases, as highlighted in [7].

It is challenging for a single model to learn all the nonlinearities in traffic data due to dynamically changing traffic conditions. To address this issue, data-driven approaches have been combined with increasing the predictive accuracy of various traffic prediction tasks and being viable alternatives to traditional learning models. For instance, the authors of [8] proposed an ensemble approach comprising extreme gradient boosting (XGB) and a gated recurrent unit (GRU) for freeway TTP. Similarly, Li et al. [9] employed XGB and a light gradient boosting machine (LightGBM) using floating car data (FCD) for urban network TTP. In another study [10], MLP and LightGBM were employed as base regressors, and a decision tree was used as a meta-regressor for OD-based TTP. Similarly, a linear regression model, a decision tree model, and the linear weighted fusion method were used as meta-regressors in [8,9,10]. However, all of these studies used the base learners’ outputs as the meta-regressors’ inputs. None of them examined the feature spaces of the base learners in combination with their decision scores for the final prediction results.

In this study, we formulated the TTP problem as a regression problem and solved it using an ensemble-based approach. We jointly exploited the feature spaces and decision scores of deep learning models, including a convolutional neural network (CNN), a multilayer perceptron (MLP), a bidirectional long short-term memory (BiLSTM) module, and a bidirectional gated recurrent unit (BiGRU), for better generalization and representation. The best-performing models’ feature spaces and decision scores (i.e., the BiLSTM and BiGRU) were hybridized and fed into a support vector regressor (SVR) to obtain the final predictions. Our results demonstrated that our proposed feature space-based BiLSTM–BiGRU approach outperformed other state-of-the-art deep learning- and ensemble-based approaches.

The main contributions of this paper can be summarized as follows:

- The proposal of a novel heterogeneous ensemble approach for travel time prediction that employed feature spaces and decision scores that were extracted from BiLSTM and BiGRU modules using hybrid learning theory and fed into an SVR for TTP;
- A principal component analysis (PCA) and deep stacked autoencoder (DSAE) enhanced the feature spaces and achieved better feature representation (using the FCD dataset. Our proposed hybridized feature space-based BiLSTM–BiGRU ensemble showed significant improvements in terms of the root mean square error (RMSE), mean absolute error (MAE), and the coefficient of determination (*R*2) compared to baseline architectures).

The remainder of this paper is organized as follows. Section 2 discusses the state-of-the-art techniques within the field of study. In Section 3, we present our proposed methodology. In Section 4, we present the results of our study. In Section 5, we present the ablation study to validate our proposed approach. Section 6 discusses the conclusion of the paper.

## 2. Related Work

Earlier studies on TTP employed segment-based and path-based approaches. In segment-based approaches, the goal was to estimate TT using a given set of routes, portions, or regions of a highway. To model segment-based TT, various algorithms have been proposed, including pattern matching, least squares minimization, hidden Markov models, gradient boosting decision trees and XGB [7,11,12,13,14]. Data fusion has also been employed before prediction to solve the limits of a single data source and increase prediction accuracy [15]. However, segment-based approaches do not consider the transition time from one link to another and link delays at intersections. To address these problems, path-based approaches have been developed [2,16,17,18]. These methods divide the entire paths into sub-paths to obtain the final predictions and then compute the TT for each sub-path using historical trajectories. Rahmani et al. [16] proposed the idea of concatenating these sub-paths to obtain the travel time of the entire path. Similarly, the pathlet dictionary was used in [17,18] for TTP. However, these approaches suffer from data sparsity, affecting their efficacy.

Data-driven approaches have become increasingly popular in the traffic forecasting area over recent years thanks to advances in data collection technologies, such as in-vehicle navigation systems, handheld devices, etc. These approaches tend to model TT end-to-end by exploiting the spatiotemporal characteristics and learning correlations in traffic data. For example, Abdollahi et al. [19] employed MLP using rich feature spaces generated by PCA, clustering analysis, and DSAE for OD-based TTP. Similarly, CNN [20], deep belief networks [21], LSTM [22], BiLSTM [23] and GRUs [24] have also been implemented for TTP in recent studies.

Data-driven approaches in the traffic forecasting domain can be categorized into OD-based approaches and trajectory-based approaches. To estimate TT, OD-based methods only consider the pick-up location, drop-off location, and departure time from historical trajectories [19,25]. Data sparsity is a problem in most OD-based systems as data that match query pick-up locations, drop-off locations, and departure times do not always exist in historical trajectories. Neighboring trips were used in [1] to handle data sparsity problems. The authors of [26] enhanced the accuracy of their model even more by first computing the distances between specific OD pairs and then predicting the TT. Xu et al. [25] combined exogenous data, such as air quality and weather, with OD features to improve model performance. Although OD-based TTP solutions are faster in computation, neglecting intermediate trajectory points causes key information to be missed, such as route variability, the number of traversed segments, the number of signals between a pick-up and drop-off location, etc. When forecasts are expanded to the network level or when driver-specific predictions are needed, the accuracy of these systems suffers. Trajectory-based approaches, on the other hand, leverage vehicle trajectories (which are ignored in OD-based prediction) to properly estimate TT [23]. Fu et al. [27] used taxi trajectory data to apply a conventional CNN and a time CNN for spatiotemporal feature learning and augmented exogenous features to improve prediction accuracy. The authors of [28,29] transformed vehicle trajectories into images and used a CNN to extract spatiotemporal features from the modified images.

Although data-driven approaches can represent and model any complex traffic condition independently, hybridization and/or ensembles of approaches could improve and boost performance even more. There has been a shift in recent studies toward these types of techniques, as cited in [30]. TTP at the corridor level was implemented in [31] by combining particle filtering and SVR. Network-wide TTP was studied using probabilistic principal component analysis, and local smoothing [32]. Zhang et al. [33] combined a CNN and LSTM to input features into a fully connected layer for TT prediction. Recent studies have also explored ensemble-based techniques in addition to hybridized models. An ensemble based on a GRU and XGB was proposed for freeway TTP in [8]. Zou et al. [10] used a decision tree model for TTP to merge the decisions of an MLP and a LightGBM. Similarly, the authors of [9] showed that model fusion incorporating LightGBM and XGB produced better results for urban road networks than standalone models. A wide–deep–recurrent learning model was proposed in [34], which combined wide (linear), deep (MLP), and recurrent (LSTM) models to predict TT. However, none of these ensemble approaches looked at the impacts of the deep learning models’ feature spaces and decision scores on TTP in a hybridized manner. In this work, we employed an SVR on the feature spaces and decision scores that BiLSTM and a BiGRU generated.

## 3. Proposed Methodology for Travel Time Prediction

**Figure 1:**
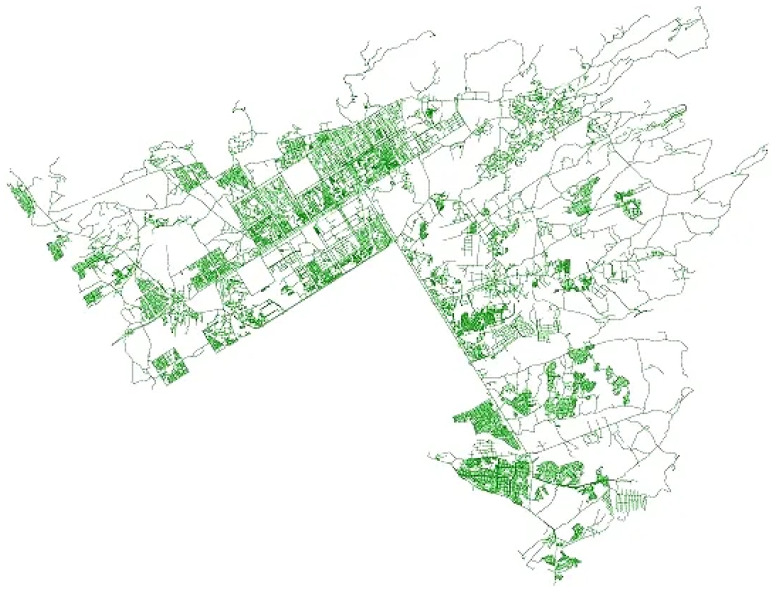
The location of the study area (Islamabad, Pakistan).

**Figure 2:**
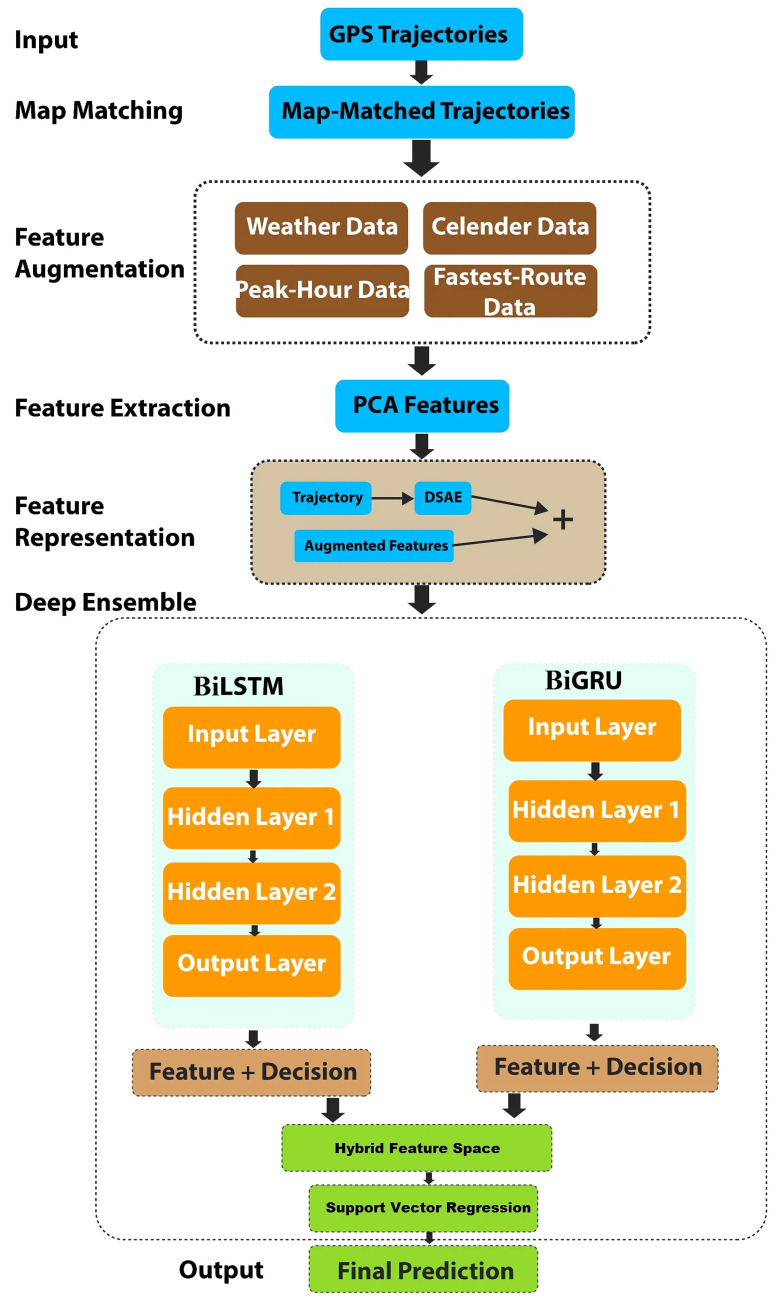
An overview of the proposed approach.

Predicting travel time is difficult since it is influenced by various factors, such as route selection, weather conditions (it takes longer to travel in bad weather conditions), time of day (peak vs. non-peak hours), etc. Ensemble-based approaches are currently the most advanced approaches for various machine learning problems. The basic idea of ensemble-based approaches is to increase the overall predictive performance of a model by addressing the inadequacies of every single approach and introducing diversity using multiple base learners. As a result of this diverse learning, a more robust model emerges that can better reflect data variations (distribution). Many methods have been utilized to integrate base learners into an ensemble model, such as voting, ensemble selection, and stacking [35]. In this study, we used a stacking-based heterogeneous ensemble approach. With an SVR acting as a meta-regressor, the feature spaces and decision scores of the BiLSTM and BiGRU were extracted using hybrid learning theory. Figure 1 depicts the study area used to test our proposed approach. A brief overview of the proposed heterogeneous ensemble is shown in Figure 2. Our proposed framework included map matching, feature augmentation, feature extraction, and representation, followed by our hybridized deep boosted feature space-based predictor.

GPS trajectories were mapped onto the OpenStreetMap network using an open-source routing machine (OSRM). Because the response times for online requests from the OSRM were so poor, we set up an offline OSRM server in a docker environment to rectify the issue. We used the parallelized batch processing and multithreading mechanism described in [36] to speed up the process even further. The algorithm presented in our previous work [37] was used to tackle challenges associated with the offroad mapping of cars and trackers at zero speed.

The weather conditions, time of day, day of the week, peak vs. non-peak hours, route choice, and other factors significantly impact travel time. We extracted and aggregated numerous geographical, temporal and weather-related features in our integrated dataset. The geographical characteristics of a trip, such as the selected route and the geographical area of the trip, have significant impacts on the TT. Using map matching, we extracted the geographic characteristics of a trip from the vehicle, such as the total distance, trajectory segments, and intersections that were crossed. Temporal characteristics also affect TT. For example, TT during peak/rush hours is very different and often much longer than during non-peak hours. We extracted the time of day, day of the week, day of the month, and month of the year features as temporal information. The weather conditions are yet another aspect that influences TT [38]. Therefore, we incorporated 18 new weather conditions (https://www.worldweatheronline.com/developer/, accessed on 7 October 2021) into our final feature set, including clear, cloudy, sunny, light rain, heavy rain, etc. Other important features that contributed to our accurate TTP included holidays, peak hours, fastest route time, and fastest route distance. Using the OSRM fastest route API (https://project-osrm.org/docs/v5.5.1/api/#route-service, accessed on 10 October 2021), the fastest route attributes that were described in [39] were extracted. The peak hours feature was determined through consultations with the Directorate of Traffic Engineering and Transportation Planning Islamabad and then validated using our data.

**Figure 3:**
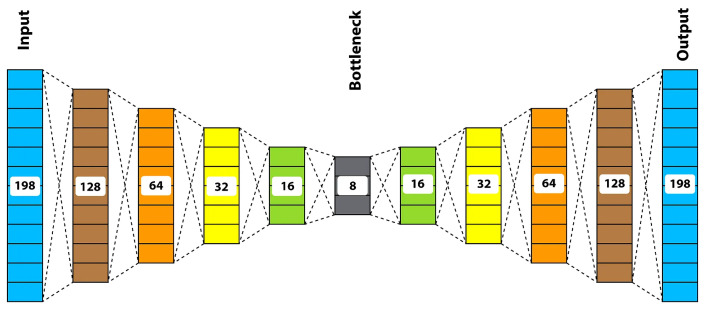
The proposed deep stacked autoencoder.

We performed a PCA on pick-up and drop-off locations to extract the top two orthogonal (uncorrelated) components to improve and boost the feature spaces [40]. The basic idea of PCA is to retain the maximum variance while reducing dimensionality. We appended these features to our feature spaces. In addition, as demonstrated in Figure 3, we used DSAE to encode trajectories and improve feature representation. The target was to extract the encoded representation of our GPS trajectories. This study encoded the trajectories into eight features (bottleneck). We combined these encoded features with other augmented feature sets to obtain the final feature set. After this data aggregation and feature representation, we performed some preprocessing to remove anomalous trips with extremely short TTs (less than 60 s) or extremely long TTs (more than 7200 s) before final experimentation. Our data included trips that ranged from 0.5 km to 60 km.

### 3.1. Scheme for Implementation

We first analyzed the feature spaces and decision scores of the state-of-the-art deep learning models separately, and then we hybridized the feature spaces with the decision scores of the best two models to produce boosted feature spaces. An SVR model was then used as a meta-model on these boosted feature spaces for the final TTP.

#### 3.1.1. Development of State-of-the-Art Deep Learning Models for TTP

We analyzed six widely used deep learning models: CNN, MLP, LSTM, GRU, BiLSTM, and BiGRU. We trained each model in an end-to-end manner, then extracted the individual models’ feature spaces and decision scores and fed them into the SVR for the final predictions. The SVR model was chosen as it was based on structural risk reduction theory. Contrary to models based on empirical risk minimization theory, the SVR tried to minimize the test errors and improve the generalization ability of the model [41]. The two best models were selected for the next phase of forming hybridized learning-based boosted feature spaces.

#### 3.1.2. Our Proposed Heterogeneous Ensemble Approach Using Hybridized Feature Spaces

In the literature, Akhtar et al. [42] employed an MLP using the intermediate layer activation of a recurrent neural network and other variants and showed promising results. Among the six models in the proposed ensemble strategy, BiLSTM and BiGRU outperformed the others and were chosen as the feature extractors. Their intermediate layer activation and decision scores were concatenated. We denoted the feature spaces and decision scores of the BiLSTM and BiGRU as $f_{l}$, $f_{g}$, $d_{l}$ and $d_{g}$, respectively. The final predictions were produced by the SVR model using the learned hybridized feature spaces of the recurrent models, as shown in Equation (1):

(1) $$\hat{y_{h}}=SVR\left(f_{l}+d_{l},f_{g}+d_{g}\right)$$

where $y_{h}$ denotes the output based on the hybridized feature spaces.

*Stacked BiLSTM: Our Proposed Base Regressor*. LSTM is a specialized type of recurrent neural network developed to address the long-term dependency issues of recurrent neural networks (RNNs) [43]. For traffic data, LSTM networks can model both segment-level information and long-term information about adjacent segments [44].

An LSTM cell comprises three gates: the input gate, forget gate, and the output gate. In this study, the computations at the three gates were carried out using Equations (2)–(4):

(2) $$i^{t}=\sigma^{s}\left(W^{i}\left[h^{t-1},x^{t}\right]+b^{i}\right)$$

(3) $$f^{t}=\sigma^{s}\left(W^{f}\left[h^{t-1},x^{t}\right]+b^{f}\right)$$

(4) $$o^{t}=\sigma^{s}\left(W^{o}\left[h^{t-1},x^{t}\right]+b^{o}\right)$$

where $i^{t}$ refers to the input gate, $f^{t}$ denotes the forget gate and $o^{t}$ represents the output gate at time *t*; $\sigma^{s}$ indicates the sigmoid activation function; $W^{i}$, $W^{f}$ and $W^{o}$ denote the weights and $b^{i}$, $b^{f}$ and $b^{o}$ denote the biases of the gates, respectively; $h^{t-1}$ denotes the hidden state/output from the previous timestamp and $x^{t}$ represents the input at the current timestamp. In this study, Equations (5) and (6) were used to compute the LSTM cell state $C^{t}$ and hidden output $h^{t}$, respectively:

(5) $$C^{t}=f^{t}⊗C^{t-1}+i^{t}⊗\mu^{t^{\prime }}\left(W^{c}\left[h^{t-1},x^{t}\right]+b^{c}\right)$$

(6) $$h^{t}=o^{t}⊗\mu^{t^{\prime }\left(C^{t}\right)}$$

where $\mu^{t^{\prime }}$ is the tanh activation function, $W^{c}$ and $b^{c}$ are the cell state’s weight, and bias and ⊗ refer to the point-wise multiplication.

**Figure 4:**
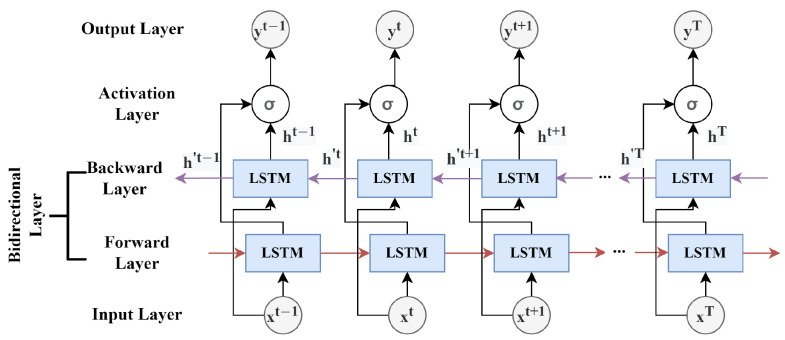
The structure of a bidirectional LSTM.

BiLSTM has recently been used to expand the learning capabilities of the LSTM model by training it twice in both the forward and backward directions. With the output layer receiving information from both past (backward) and future (forward) instances at the same time, the prediction accuracy can be improved, as shown in [45]. The structure of a BiLSTM is depicted in Figure 4. In this study, we employed a two-layered BiLSTM as one of our base regressors for travel time prediction.

*Stacked BiGRU: Our Proposed Base Regressor*. A GRU is another improved variant of an RNN, which has a simpler architectural design that consists of two gates (i.e., an update gate and a reset gate) as opposed to the three gates of LSTM [46]. Due to the simplified architecture, fewer parameters are needed to train in GRUs, which increases the model’s overall efficiency. The input and forget gates of LSTM are replaced by the update gate in GRUs.

In this study, Equations (7)–(10) were used to govern the flow of information inside the GRU cell:

(7) $$r^{t}=\sigma^{s}\left(W^{r}x^{t}+U^{r}h^{t-1}\right)$$

(8) $$u^{t}=\sigma^{s}\left(W^{u}x^{t}+U^{u}h^{t-1}\right)$$

(9) $$h^{\prime^{t}}=\mu^{t^{\prime }}\left(Wx^{t}+r^{t}⊙Uh^{t-1}\right)$$

(10) $$h^{t}=u^{t}⊙h^{t-1}+\left(1-u^{t}⊙h^{\prime^{t}}\right)$$

where $r^{t}$ and $u^{t}$ denote the reset gate and the update gate, $h^{\prime^{t}}$ and $h^{t}$ refer to the current and final memory contents at time t, $\mu^{t^{\prime }}$ and $\sigma^{s}$ are the tanh and sigmoid activation functions. $W^{u}$ and $U^{u}$ are the weights of the respective gates, ⊙ represents the element-wise multiplication, $x^{t}$ denotes the current input, and $h^{t-1}$ denotes the hidden state or the output from the previous timestamp.

**Figure 5:**
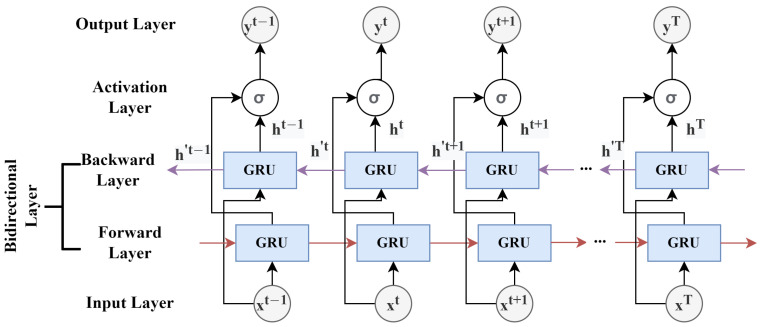
The structure of a bidirectional GRU model.

BiGRUs strengthen the predictive power of GRUs by using forward and backward passes during training. Compared to the GRU model, BiGRUs consider both previous and future values when making predictions [47]. We employed a two-layer BiGRU model in this study. The structure of a BiGRU model is depicted in Figure 5.

In this study, the computations at the forward hidden layer, backward hidden layer, and the output layer in both the BiLSTM and BiGRU were carried out by Equations (11)–(13). The difference between this model and our model lies in the fundamental components used in the forward and hidden layers, i.e., LSTM for BiLSTM and a GRU for BiGRU.

(11) $$h^{f_{t}}=f\left(W^{f_{i}}x^{t}+W^{f_{h}}h^{t-1}\right)$$

(12) $$h^{b_{t}}=f\left(W^{b_{i}}x^{t}+W^{b_{h}}h^{\prime t+1}\right)$$

(13) $$o^{t}=g\left(W^{f_{o}}h^{f^{t}}+W^{b_{o}}h^{b_{t}}\right)$$

where $h^{f_{t}}$, $h^{b_{t}}$ and $o^{t}$ denote the state variables of the forward hidden layer, backward hidden layer and the output layer, respectively, $W^{f_{i}}$, $W^{f_{h}}$, $W^{f_{o}}$, $W^{b_{i}}$, $W^{b_{h}}$ and $W^{b_{o}}$ represent the weights of the hidden input layer, hidden layer and hidden output layer in the forward and backward directions, respectively, and *f* and *g* denote the activation functions.

## 4. Experimental Results

This section describes the data, followed by an explanation of the models that were used to analyze the data and their results.

### 4.1. Dataset

We gathered and compiled a real-world anonymized FCD dataset for 2019 using data from a tracking firm in Islamabad, Pakistan.

In this study, we used data from March to October 2019. The dataset contained events captured by 2895 unique tracker IDs over the specified period. A GPS chipset (U-Blox EVA-M8M) and a GSM modem (Quectel M95) were used to mount the tracker units. Table 1 provides detailed statistics about the dataset. This study used data from 6:00 a.m. to 11:00 p.m., including peak and non-peak hours.

**Figure 6:**
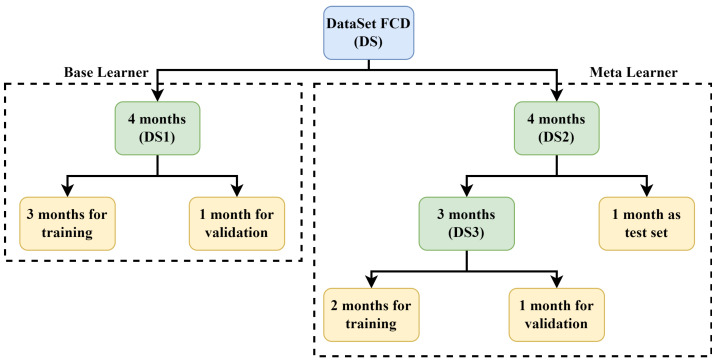
The data distribution between the base regressor and meta-regressor.

Figure 6 shows the data distribution of our final feature set between the base regressor and the meta-regressor.

For the base learners, we used four months’ data (DS1): three months’ data was used for training, and the remaining one month’s data was used for validation. For the meta-learner, four months’ data (DS2) was used. The meta-learner was trained and validated using data from the previous three months (DS3). Finally, one month’s data was used as a testing set to evaluate the proposed approach’s generalization and report our results.

### 4.2. Performance Metrics

We used three evaluation techniques to assess our proposed model and baseline techniques: RMSE, MAE, and $R^{2}$. We let TT_i denote the actual travel time and $\hat{TT_{i}}$ indicate the predicted travel time, then RMSE could be expressed as in Equation (14):

(14) $$RMSE=\sqrt{\frac{1}{n}\sum_{i=1}^{n}{\left(\hat{TT_{i}}-TT_{i}\right)}^{2}}$$

MAE refers to the average absolute error out of actual and estimated values and was calculated using Equation (15):

(15) $$MAE=\frac{1}{n}\sum_{i=1}^{n}\left|\hat{TT_{i}}-TT_{i}\right|$$

$R^{2}$ indicates how much of a variation is learned by a model and was calculated using Equation (16):

(16) $$R^{2}=1-\frac{\sum_{i=1}^{n}|(\hat{TT_{i}}-TT_{i})|}{\sum_{i=1}^{n}|(\hat{TT_{i}}-TT_{m})|}$$

where TT_m refers to the mean travel time. These equations were taken from [48]. For the best prediction, the ideal values for RMSE and MAE were zero (or close to zero), and the ideal value for $R^{2}$ was close to one.

### 4.3. Experimental Settings

We ran all the simulations using Keras (2.3.1), based on Tensor Flow (2.1.0) and Python 3.7.16. All models were trained using an NVIDIA GeForce GTX 1070 Ti-equipped machine.

### 4.4. Baseline Techniques

We tested six state-of-the-art deep learning architectures as there was no prior research on our data: MLP [49], CNN [50], LSTM [44], GRU [46], BiLSTM [51] and BiGRU [47]. Furthermore, we also implemented three related ensemble approaches [8,9,10] using our dataset and compared the results to those from our proposed heterogeneous ensemble approach.

### 4.5. Hyperparameter Settings

**Figure 7:**
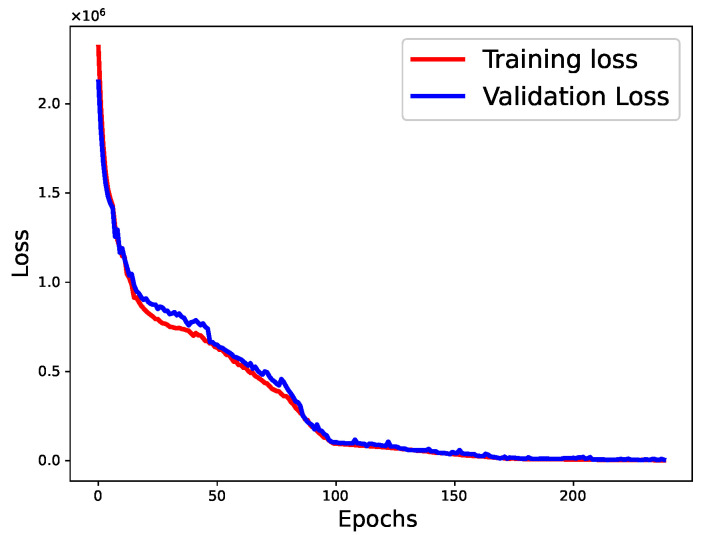
The loss curves of the BiGRU (training and validation data).

**Figure 8:**
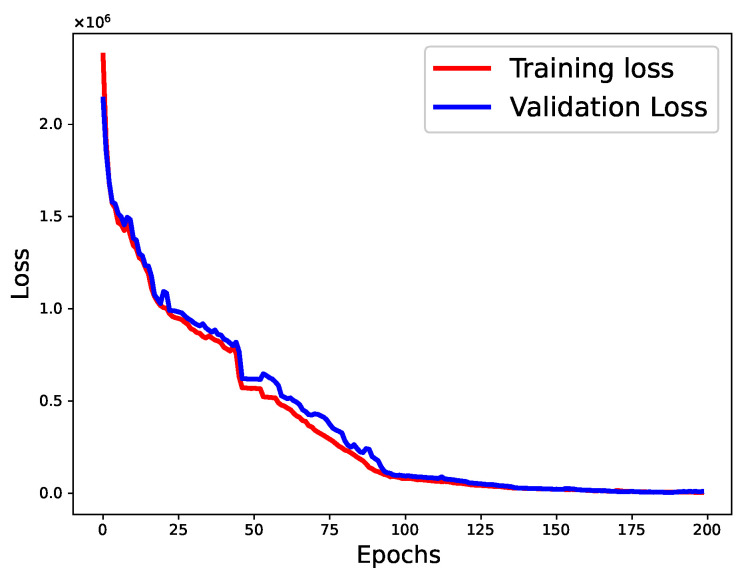
The loss curves of the BiLSTM (training and validation data).

The parameter settings for our baseline NNs are presented in Table 2. These values were obtained using the trial-and-error method. After several experimental runs, we obtained the optimal values for each parameter of the models, as listed in Table 2. We varied the learning rate, the number of hidden layers, the number of neurons in each hidden layer, and the batch size of our base regressors. The activation function and optimizer were set to “ReLU” and “Adam”, respectively. At first, we conducted the experiment for 50 epochs and observed the overfitting of the model. To address this, we used early stopping and dropout regularization with a dropout ratio of 0.2; we ran the experiment for 500 epochs. Holdout cross-validation was used to validate the results of our proposed approach (Figure 6). The loss curves of the BiGRU and BiLSTM utilizing the training and validation data are shown in Figure 7 and Figure 8, respectively. Unlike the baseline techniques, our proposed approach involved a machine learning-based meta-model (SVR), which demonstrated pseudo-random behavior (as with other machine learning models). Therefore, we ran the experiment 10 times with the optimal parameters and reported the confidence intervals to prove the robustness of our approach.

### 4.6. Performance Evaluation of the State-of-the-Art Deep Learning Models

In this section, we present the results of the individual deep learning models as feature extractors (feature spaces and decision scores) for the SVR using the overall data (i.e., the dataset included both weekday and weekend data). The results are summarized in Table 3.

The CNN was not appropriate for our data, as shown in Table 3. It is due to CNN’s failure to account for temporal factors when making a prediction. The RMSE was reduced to 135.85 s, and the MAE was decreased to 28.85 s by the MLP, but both were still very high for real-world applications. Compared to these conventional models, the specialized time-series models (LSTM, GRU, and their two variants, BiLSTM and BiGRU) performed significantly better using the same data. The RMSE values of the GRU, LSTM, BiGRU, and BiLSTM were reduced to 71.12, 70.33, 63.62, and 62.48 s, respectively. As can be seen from these results, the error metrics for the BiGRU and BiLSTM were significantly lower compared to those for the GRU and LSTM. The reason for this was that these specialized variants took into account past observations as well as future observations at the same time while making predictions, unlike the LSTM and GRU, which were unidirectional models that only considered past observations in their predictions.

### 4.7. Performance Evaluation of Our Proposed Heterogeneous Ensemble Approach Using the Overall Data

**Figure 9:**
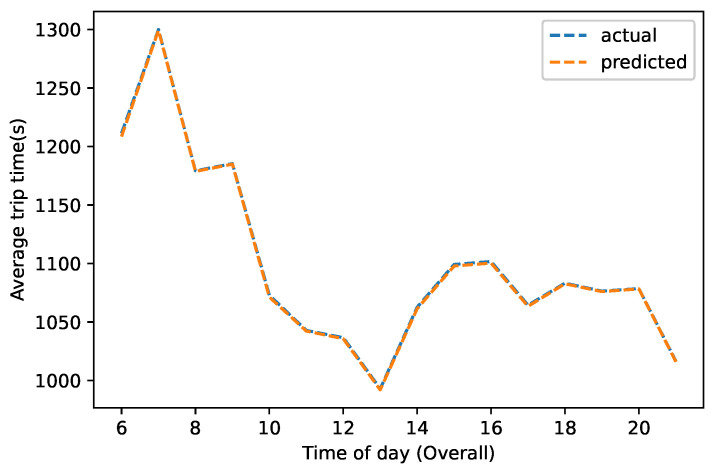
The actual vs. predicted normalized travel time using our proposed heterogeneous BiLSTM–BiGRU-based ensemble approach.

The BiLSTM and BiGRU performed better as feature extractors and outperformed the CNN, MLP, GRU, and LSTM, as discussed in Section 4.6. The creation of hybridized feature spaces by combining the feature spaces and decision scores of these two specialized recurrent learning models could increase the overall performance [42]. As a result, we created hybridized deep boosted feature spaces by combining the feature spaces and decision scores of these two benchmark specialized time-series models. The results were further improved when these boosted feature spaces were fed into the SVR for the final predictions, as shown in Table 4. The best results in terms of RMSE (53.87±3.50), MAE (12.22±1.35), and $R^{2}$ (0.99784±0.00019) were obtained by hybridizing the feature spaces with the decision scores of the BiLSTM and BiGRU models (i.e., hybridized BiLSTM–BiGRU). In our data, as summarized in Table 1, the average distance was approximately 6 km, and the mean travel time was 1109.50 s. In this context, the RMSE value of 53.87 s was a promising result. We could deduce from these findings that when these models were employed together for a task, they complemented each other when correctly tuned. Additionally, using these models’ feature spaces and decision scores in conjunction with other classical models could improve performance. Using our proposed approach, Figure 9 depicts the actual vs. predicted normalized travel time at different times of the day, from 6:00 a.m. to 11:00 p.m.

In addition, we conducted two further experiments to demonstrate the generalizability of our proposed heterogeneous ensemble approach by investigating the impacts of weather features and testing our model using only weekday data. Only a minor reduction in model performance was reported in each instance. The details are provided in the following subsections.

#### 4.7.1. Impact of Weather on Model Performance

**Figure 10:**
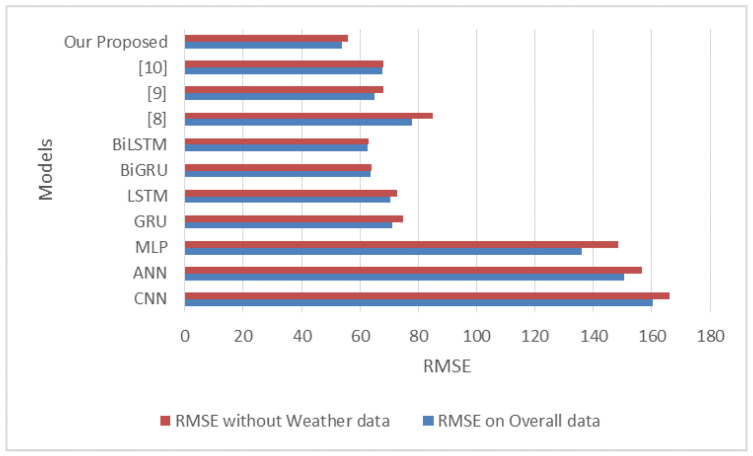
A comparison of the RMSE values with and without the weather data.

Weather conditions are an important exogenous factor that can affect travel time. We assessed the performance of our proposed ensemble and the baseline techniques using the overall data without weather features to demonstrate the importance of complementing weather conditions and traffic data. To see how weather data affected the overall performance, we removed 18 weather features from the data. The results of this experiment are summarized in Table 5. The performance of the deep learning models (CNN, MLP, GRU, LSTM, BiLSTM, and BiGRU) and the ensemble model was degraded when the weather data was removed. The RMSE value produced by our proposed heterogeneous ensemble increased to 55.71±5.41 s, indicating the considerable effect of weather features on overall TT prediction. The RMSE values that our proposed hybridized BiLSTM produced–BiGRU ensemble and the baseline techniques are shown in Figure 10.

#### 4.7.2. Impact of Using Weekday Data Only on Model Performance

**Figure 11:**
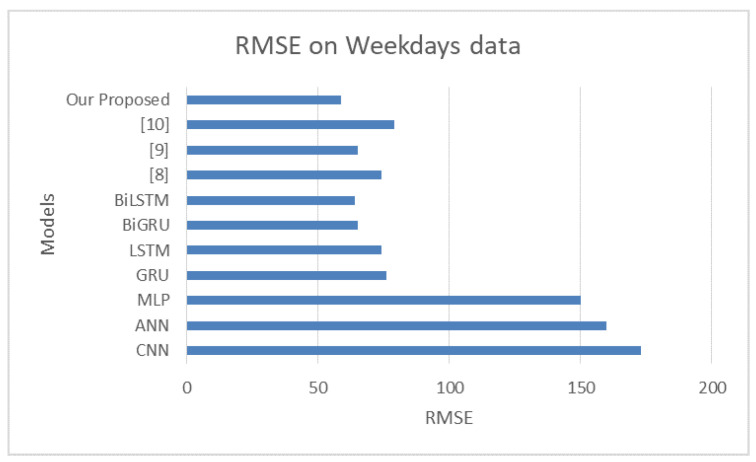
A comparison of the RMSE values with and without the weekend data.

The results of this experiment are presented in Table 6. The performance of the proposed approach was only slightly degraded by omitting the weekend data, and the RMSE value increased from 53.87±3.50 s to 56.70±4.91 s. The RMSE values that our proposed hybridized BiLSTM produced–BiGRU ensemble and the baseline techniques are shown in Figure 11.

The performance of the models from [8,9,10] deteriorated slightly when the weekend data was omitted. The ensemble approach proposed in [8] produced RMSE and MAE values of 74.11 and 31.94, respectively. The ensemble approach presented in [10] had RMSE and MAE values of 78.87 and 30.26, respectively. Similarly, the RMSE and MAE values produced by the ensemble approach proposed in [9] were 65.24 and 23.78, respectively.

### 4.8. Performance Evaluation of Our Proposed Heterogeneous Ensemble Approach and the Reported Ensemble Approaches Using the Overall Data

Our proposed boosted feature space-based heterogeneous ensemble approach performed significantly better than the existing ensemble baseline techniques described in the literature, as shown in Table 7. The authors of [8] combined the scores of a gradient boosting decision tree-based ensemble (XGBoost) with those of a GRU and reported RMSE and MAE values of 77.75 and 33.90, respectively. Similarly, the authors of [10] combined the scores of a LightGBM (another lightweight gradient boosting decision tree model) with those of a deep learning model (MLP) and reported RMSE and MAE values of 67.71 and 22.78, respectively. Moreover, the authors of [9] combined the scores of two decision tree-based ensemble models to improve the overall performance. In this study, the ensemble of the LightGBM and XGBoost produced RMSE and MAE values of 65.05 and 23.34, respectively; however, none of these approaches hybridized the feature spaces and decision scores of deep learning models with the capabilities of ML models.

## 5. Ablation Study

We carried out an ablation study to demonstrate the impacts of feature augmentation, feature extraction, and representation within our proposed approach. We removed the feature augmentation, feature extraction, and representation stages in our baseline experiment. The impact of each feature/module on the outcome is shown in Table 8. It was evident that adding exogenous features, such as weather, calendar dates, peak hours and the fastest route, to the PCA features and encoded features significantly improved the overall performance: the RMSE improved from 63.62±7.77 s to 53.87±3.50 s, the MAE improved from 22.07±3.98 s to 12.22±1.35 s and the $R^{2}$ value increased from 0.99708±0.00047 to 0.99784±0.00019.

By using DSAE to compress the GPS trajectories into eight encoded features, we greatly reduced the dimensionality of our final feature set, which further enhanced the performance of the baseline model. Deep autoencoders have been widely adopted in data/feature compression techniques in various domains [52]. A typical deep stacked autoencoder consists of an encoder and a decoder with multiple layers each and a coded layer (also called a bottleneck), as illustrated in Figure 3. The basic idea of these autoencoders (AEs) is first to learn the coded representation from the input using the encoder and then to reconstruct the input from the coded representation using the decoder. This coded representation after training contains the maximum information needed to reproduce the input in a lower dimensional space. Similarly, the projection of pick-up and drop-off locations using the PCA improved our model performance. To further validate the impact of DSAE and PCA (as reported in Table 8), we computed the importance of these features using a well-known feature importance technique called mutual information regression, which measures the information gain of features concerning the output variables. These measurements were calculated using Equation (17):

(17) MI(F;T)=E(F)−E(F|T)

**Figure 12:**
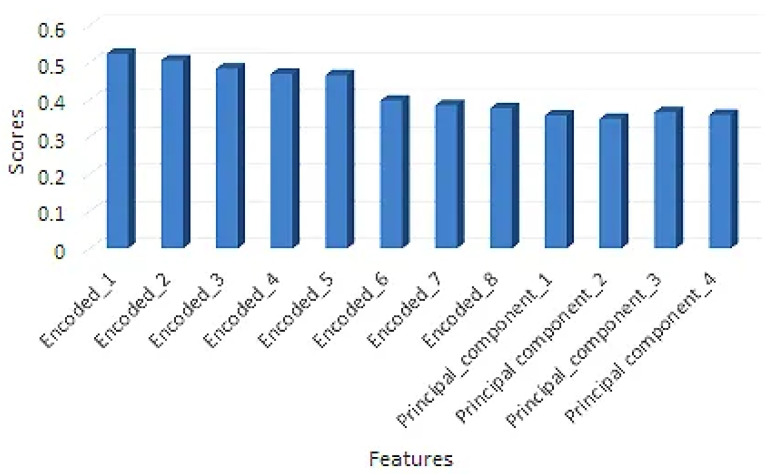
The DSAE and PCA feature importance validation using mutual information regression.

The validation results are reported in Figure 12, which shows a good correlation between the transformed features and the output (travel time). The outcome ranged from 0 to *∞*. Higher values suggested a stronger correlation between the features and the target and were used in the final feature set. In this study, we used DSAE for feature encoding; other AE variants, such as denoising AEs and variational AEs, could further enhance these results. In addition, the Huber loss function could be used instead of the mean square error, which uses a delta parameter to control the weight updates [53].

## 6. Conclusions

Travel time prediction is one of the most challenging issues in the mobility-related applications of smart cities. We developed a novel heterogeneous ensemble approach that was based on a hybridized feature learning strategy. FCD data were augmented with various endogenous and exogenous data that affected travel time, including peak hours, weather conditions, calendar dates, etc. Moreover, we extracted PCA features and encoded trajectories using an autoencoder to enhance the feature spaces and reduce data dimensionality. These data were fed into six state-of-the-art deep learning models: CNN, MLP, LSTM, GRU, BiLSTM, and BiGRU. Then, their feature spaces and decision scores were analyzed using an SVR as a meta-regressor for TTP. The feature spaces and decision scores of the two best-performing models (BiLSTM and BiGRU) were then concatenated to generate hybridized deep boosted feature spaces. The SVR was employed for the final predictions in these hybridized feature spaces. We achieved an RMSE value of 53.87±3.50, an MAE value of 12.22±1.35 and a coefficient of determination of 0.99784±0.00019 using our proposed hybridized learning-based heterogeneous ensemble. We also performed an ablation study to test the robustness of our proposed approach. Our proposed hybridized BiLSTM–BiGRU model yielded better performance than the selected baseline techniques. The proposed method was distinguished from the other ensemble approaches based on their base regressors’ decision scores. As our proposed approach involved tuning base regressors and meta-regressors in two stages, the training required a little more time than the baseline techniques; however, this was negligible due to the availability of GPU-based machines. This study did not explore other SVR kernels, such as radial basis function, polynomial, etc. Furthermore, other AEs variants, such as denoising AEs and variational AEs, were also not explored in this study. In the future, we plan to investigate transformer networks using the same dataset. We also plan to evaluate the performance of graph-based neural networks using the same dataset.

## Figures and Tables

**Table 1:** A summary of the FCD dataset.

Attribute	Value
Trajectory Count	724,402
Area	220 km^2^
Sampling Rate	15 s–45 s
Travel Time Mean	1109.50 s
Travel Time Standard Deviation	1173.51 s
Travel Distance Mean	5986.96 m
Travel Distance Standard Deviation	6732.36 m

**Table 2:** The optimal parameter settings of the baseline techniques.

Model	Parameter	Value
CNN	Convolution Layers	2
	Max-Pooling Layers	1
	Filter Size	(64,32)
	Kernel Size	3
	Pool Size	3
	Activation	ReLU
	Optimizer	Adam
	Learning Rate	0.0001
MLP	Layers	2
	Neurons	(64,64)
	Activation	ReLU
	Learning Rate	0.001
	Optimizer	Adam
	Batch Size	256
LSTM	Layers	2
	Neurons	(64,64)
	Activation	ReLU
	Learning Rate	0.001
	Optimizer	Adam
	Batch Size	128
GRU	Layers	2
	Neurons	(64,64)
	Activation	ReLU
	Learning Rate	0.001
	Optimizer	Adam
	Batch Size	128
BiLSTM	Layers	2
	Neurons	(64,64)
	Activation	ReLU
	Learning Rate	0.001
	Optimizer	Adam
	Batch Size	128
BiGRU	Layers	2
	Neurons	(64,64)
	Activation	ReLU
	Learning Rate	0.001
	Optimizer	Adam
	Batch Size	128
SVR	Kernel	Linear
	C	1.0
	Maximum Iterations	1000

**Table 3:** The performance evaluation of the state-of-the-art deep learning models.

Model	RMSE (s)	MAE (s)	R^2^ (%)
CNN	160.31	62.63	0.980180
MLP	135.85	28.85	0.986653
GRU	71.12	24.09	0.996805
LSTM	70.33	23.93	0.996887
BiGRU	63.62	19.38	0.997215
BiLSTM	62.48	17.41	0.997553

**Table 4:** The performance evaluation of our proposed heterogeneous ensemble approach using the overall data.

Model	RMSE(s)	MAE(s)	$R^{2}$(%)
**Proposed Hybridized BiLSTM–BiGRU Model**	53.87 ± 3.50	12.22 ± 1.35	0.99784 ± 0.00019

**Table 5:** The results from the experiment on the impact of weather conditions on TTP.

Model	RMSE (s)	MAE (s)	$R^{2}$ (%)
CNN	166.21	64.52	0.980022
MLP	148.67	31.14	0.984015
GRU	74.76	26.66	0.996619
LSTM	72.93	25.87	0.996727
BiGRU	63.98	19.62	0.997187
BiLSTM	63.12	18.48	0.997498
GRU + XGB [8]	84.96	33.96	0.994780
LightGBM + XGB [9]	67.91	24.99	0.996665
MLP + LightGBM [10]	67.95	24.91	0.996661
**Proposed Hybridized BiLSTM–BiGRU Model**	55.71 ± 5.41	13.29 ± 2.31	0.99767 ± 0.00088

**Table 6:** The results from the experiment on the impact of omitting weekend data on TTP.

Model	RMSE (s)	MAE (s)	$R^{2}$ (%)
CNN	173.01	65.51	0.976142
MLP	150.11	34.12	0.983802
GRU	75.99	27.79	0.996505
LSTM	74.03	26.39	0.996645
BiGRU	65.04	20.75	0.997019
BiLSTM	64.08	18.79	0.997325
GRU + XGB [8]	74.11	31.94	0.996045
LightGBM + XGB [9]	65.24	23.78	0.996935
MLP + LightGBM [10]	78.87	30.26	0.995521
**Proposed Hybridized BiLSTM–BiGRU Model**	56.70 ± 4.91	15.06 ± 2.15	0.99754 ± 0.00085

**Table 7:** Performance comparison of our proposed heterogeneous and reported ensemble approaches using the overall data.

Model	RMSE (s)	MAE (s)	$R^{2}$ (%)
GRU + XGB [8]	77.75	33.90	0.995629
LightGBM + XGB [9]	65.05	23.34	0.996940
MLP + LightGBM [10]	67.71	22.78	0.996685
**Proposed Hybridized BiLSTM–BiGRU Model**	53.87 ± 3.50	12.22 ± 1.35	0.99784 ± 0.00019

**Table 8:** The ablation study of our proposed heterogeneous ensemble approach.

Model	RMSE (s)	MAE (s)	*R*^2^ (%)
Baseline	63.62±7.77	22.07±3.98	0.99708±0.00047
+ DSAE	59.67±4.64	18.10±2.48	0.99744±0.00029
+ PCA Features	58.34±4.08	17.32±1.94	0.99756±0.00025
+ Weather Data	56.93±2.85	15.77±1.09	0.99767±0.00019
+ Calendar Dates	56.04±2.13	14.49±0.98	0.99770±0.00043
+ Peak Hours	55.68±2.18	13.87±1.98	0.99780±0.00022
+ Fastest Route	53.87±3.50	12.22±1.35	0.99784±0.00019

## Data Availability

The data that are presented in this study are available upon request from the authors.

## Abbreviations

A list of the abbreviations and symbols (Abbr.) that are used in this paper.

**Abbr.**	**Description**	**Abbr.**	**Description**
ITS	Intelligent transportation system	$f_{l}$	LSTM feature space
TTP	Travel time prediction	$f_{g}$	GRU feature space
ATIS	Advanced traveler information system	$d_{l}$	LSTM decision space
GPS	Global positioning system	$d_{g}$	GRU decision space
OD	Origin–destination	$y_{h}$	Final output
GRU	Gated recurrent unit	$i^{t}$	Input gate
XGB	Extreme gradient boosting	$f^{t}$	Forget gate
LightGBM	Light gradient boosting machine	$o^{t}$	Output gate
FCD	Floating car data	$\sigma^{s}$	Sigmoid activation function
MLP	Multilayer perceptron	$W^{i}$	Input gate weight
CNN	Convolutional neural network	$W^{f}$	Forget gate weight
BiLSTM	Bidirectional long short-term memory	$W^{o}$	Output gate weight
BiGRU	Bidirectional gated recurrent unit	$b^{i}$	Input gate bias
SVR	Support vector regressor	$b^{f}$	Forget gate bias
RMSE	Root mean square error	$b^{o}$	Output gate bias
MAE	Mean absolute error	$h^{t-1}$	Hidden state of prior timestamp
R2	Coefficient of determination	$x^{t}$	Current input
PCA	Principal component analysis	$C^{t}$	Cell state
DSAE	Deep stacked autoencoder	$h^{t}$	Hidden output
LSTM	Long short-term memory	$\mu^{t^{\prime }}$	Tanh activation function
OSRM	Open-source routing machine	$W^{c}$	Cell state weight
$u^{t}$	Update gate	$b^{c}$	Cell state bias
${h^{\prime }}^{t}$	Current memory content time (t)	$r^{t}$	Reset gate
$h^{t}$	Final memory content time (t)	$W^{r}$, $U^{r}$	Reset gate weight
$W^{u}$, $U^{u}$	Update gate weight	⊙	Element-wise multiplication
$h^{f_{t}}$	Hidden state variable (Forward)	$h^{b_{t}}$	Hidden state variable (Backward)
$o^{t}$	Output layer state variable	$W^{f_{o}}$	Hidden output weight (Forward)
$W^{b_{i}}$	Hidden input weight (Backward)	$W^{f_{i}}$	Hidden input weight (Forward)
$W^{b_{h}}$	Hidden weight (Backward)	$W^{f_{h}}$	Hidden weight (Forward)
$W^{b_{o}}$	Hidden output weight (Backward)	f	Hidden layer activation
g	Output layer activation	TT_i	Actual travel time
$\hat{TT_{i}}$	Predicted travel time	TT_m	Mean travel time
MI	Mutual information	E	Entropy
F	Feature	T	Target
